# Predicting finite-temperature properties of crystalline carbon dioxide from first principles with quantitative accuracy†
†Electronic supplementary information (ESI) available: Additional methodological details, optimized structures, and tables of the predicted properties. See DOI: 10.1039/c5sc03014e


**DOI:** 10.1039/c5sc03014e

**Published:** 2015-09-29

**Authors:** Yonaton N. Heit, Kaushik D. Nanda, Gregory J. O. Beran

**Affiliations:** a Department of Chemistry , University of California , Riverside , California 92521 , USA . Email: gregory.beran@ucr.edu ; Tel: +1-951-827-7869

## Abstract

Molecular crystal structures, thermodynamics, and mechanical properties can vary substantially with temperature, and predicting these temperature-dependencies correctly is important for many practical applications in the pharmaceutical industry and other fields. However, most electronic structure predictions of molecular crystal properties neglect temperature and/or thermal expansion, leading to potentially erroneous results. Here, we demonstrate that by combining large basis set second-order Møller–Plesset (MP2) or even coupled cluster singles, doubles, and perturbative triples (CCSD(T)) electronic structure calculations with a quasiharmonic treatment of thermal expansion, experimentally observable properties such as the unit cell volume, heat capacity, enthalpy, entropy, sublimation point and bulk modulus of phase I crystalline carbon dioxide can be predicted in excellent agreement with experiment over a broad range of temperatures. These results point toward a promising future for *ab initio* prediction of molecular crystal properties at real-world temperatures and pressures.

## Abstract 2

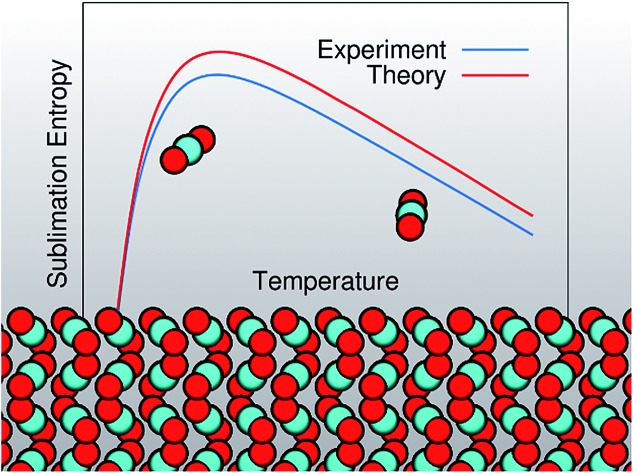
The temperature-dependence of the crystalline carbon dioxide (phase I) structure, thermodynamics, and mechanical properties are predicted in excellent agreement with experiment over a 200 K temperature range using high-level electronic structure calculations.

## 1 Introduction

Moving beyond 0 K to predict molecular crystal structures and properties at finite temperatures represents the next frontier in modeling organic materials. Instead of predicting a multitude of potential crystal polymorphs at 0 K, can we tell a pharmaceutical chemist if the desired drug formulation is thermodynamically favored at room temperature? Or can we predict the polymorphic phase diagram over a broad range of temperatures and pressures? The unexpected appearance of a new, more stable polymorph of a drug can have dire consequences for its stability or bioavailability, sometimes even forcing the drug's removal from the market until a new formulation can be developed.^1^–^3^

When manufacturing a drug tablet, the rapid decompression which occurs immediately after compaction of the drug powder can lead to a variety of defects or even catastrophic failure of the tablet.^4^ Mechanical properties like the Young's modulus and the bulk modulus of a molecular crystal provide important insights into the bulk behavior of pharmaceutical powders during the tabletting process.^5^–^7^ Given that such mechanical properties can exhibit sizable temperature dependence, can we predict them at the relevant temperatures?

Crystal structure prediction has undergone rapid advances over the past several decades, with a number of successful predictions in the blind tests of crystal structure prediction,^8^–^13^ improved optimization algorithms^14^–^22^ for identifying stable crystal packing motifs, and major advances in dispersion-corrected density functional theory (DFT)^23^–^31^ and fragment-based electronic structure methods^32^–^45^ that enable the routine application of high-accuracy quantum mechanical methods to organic crystals. Molecular crystal lattice energies can now be predicted to within sub-kJ mol^–1^ accuracy in certain cases,^39^ or within a few kJ mol^–1^ more routinely.^25^,^26^,^30^,^31^,^41^,^46^

The next generation of molecular crystal modeling needs to move beyond 0 K lattice energies and structures, and predict crystal structures and properties at the finite temperatures and pressures where most real-world experimental applications occur. Progress in this direction has already been made. For example, DFT studies of high-pressure molecular crystal phases have become routine, and Hirata and co-workers have recently predicted the phase boundary for phase I and phase III carbon dioxide with second-order Møller–Plesset perturbation theory (MP2).^33^,^34^ They have also used similar calculations to simulate various properties and spectroscopic features in ice,^47^,^48^ carbon dioxide,^35^,^49^ and other systems.^33^ Reilly and Tkatchenko used harmonic free energy estimates with many-body dispersion-corrected density functional theory to rationalize the experimental preference for form I aspirin over form II.^50^ However, most such studies, particularly those based on *ab initio* methods beyond DFT, rely on structures optimized without consideration of temperature. The volume of a molecular crystal unit cell often expands by several percent between 0 K and room temperature, with substantial impacts on many crystal properties.

Capturing these finite temperature effects is challenging. Molecular dynamics and Monte Carlo simulations provide a conceptually straightforward means of accessing these finite-temperature properties that has proved effective for studying organic crystal free energies/phase diagrams,^19^,^20^,^51^–^54^ and nucleation/growth^55^–^58^ at the force field level. However, achieving the requisite accuracy in larger, non-rigid molecules with force fields remains a major challenge. On the other hand, the comparatively high computational cost of more accurate electronic structure methods makes extensive configurational sampling infeasible in most cases.

Instead, we demonstrate here that coupling large-basis second-order MP2 and coupled cluster singles, doubles and perturbative triples (CCSD(T)) electronic structure calculations with the quasi-harmonic approximation enables one to predict a wide variety of properties of crystalline carbon dioxide (phase I) with unprecedented accuracy. The quasi-harmonic approximation has a long-history in materials modeling, but to our knowledge, this study represents the first time it has been combined with electronic structure calculations that approach the *ab initio* limit for molecular crystals.

Carbon dioxide is much smaller than typical organic compounds, of course. It also exhibits weaker many-body interactions than many larger and/or polar molecules. Nevertheless, it makes an excellent test case for several reasons: a wealth of experimental data exists against which the predictions can be tested, its small molecular size makes it feasible to assess the accuracy that can be obtained with calculations which approach the *ab initio* limit, and it has also been the subject of many earlier DFT^59^–^62^ and smaller-basis MP2 studies.^34^,^35^,^49^

We show that extrapolated complete basis set MP2 and CCSD(T) calculations predict the crystal volume within 2%, the heat capacity within 0.2*R* (<5% for *T* = 50–190 K), the sublimation enthalpy within 1.5 kJ mol^–1^, and the sublimation entropy within 2 J mol^–1^ K^–1^ (2%), all over a temperature range spanning 200 K. CCSD(T) predicts the sublimation point of dry ice (194.7 K) to within 6 K. In contrast to previous difficulties in modeling the bulk modulus of crystalline CO_2_,^35^ we predict both its magnitude and temperature dependence in excellent agreement with experiment. Overall, the ability to achieve quantitative accuracy for a broad spectrum of molecular crystal properties in phase I carbon dioxide provides much cause for optimism in the future extension of finite-temperature predictions to larger, more chemically interesting species.

## 2 Theory and methods

The structure of phase I carbon dioxide at a given temperature *T* and pressure *P* was predicted by minimizing the Gibbs free energy *G*(*T*, *P*) = *U*_el_ + *PV* + *F*_vib_(*T*) with respect to both the atomic positions in the unit cell and the unit cell parameters. Here, *U*_el_ is the internal electronic energy, *PV* is the pressure–volume contribution, and *F*_vib_ represents the Helmholtz vibrational free energy contribution. The phonon frequencies were estimated as a function of the crystal volume using the quasiharmonic approximation (QHA).

The electronic energy and phonons were computed using the fragment-based hybrid many-body interaction (HMBI) model,^40^,^41^,^63^,^64^ which allows one to perform high-level MP2 or coupled cluster calculations on periodic systems like molecular crystals with reasonable computational cost. HMBI decomposes the intermolecular interactions in a crystal according to a many-body expansion,

1 *U*_el_ = *E*QM1-body + *E*QMSR 2-body + *E*MMLR 2-body + *E*MMmany-body

The important intramolecular (1-body) and short-range pairwise (SR 2-body) interactions were treated with quantum mechanics (QM), while the generally weaker long-range pairwise (LR 2-body) and many-body contributions in eqn (1) were approximated with the Amoeba polarizable molecular mechanics (MM) force field. In practice, the short-range 2-body QM treatment includes interactions involving molecules in the unit cell and in nearby periodic image cells, while the MM terms capture the long-range periodicity of the crystal *via* Ewald summation.

The harmonic phonons used to evaluate *F*_vib_ were computed on a 3 × 3 × 3 Monkhorst–Pack grid in a 3 × 3 × 3 supercell. Fragment methods like HMBI enable lattice dynamic calculations at many *k* points in reciprocal space with trivial additional effort beyond the *Γ*-point-only phonons.^32^,^65^ The Grüneisen parameters were computed *via* finite difference.^26^

Substantial computational savings were obtained by exploiting the *Pa*3 space group symmetry of phase I CO_2_ throughout.^66^ Symmetry reduces the number of two-body dimer calculations required from ∼100 to 5–9 (depending on the pressure). It also reduces the number of degrees of freedom in the geometry optimization from 42 to two: the lattice constant *a* and the C=O bond length.

All QM contributions were calculated with either density-fitted MP2 (ref. 67–70) or CCSD(T)^71^,^72^ in the Dunning aug-cc-pVXZ basis sets (abbreviated as aXZ here)^73^,^74^ using Molpro 2012.^75^,^76^ A counterpoise correction for basis set superposition error^77^ was employed for each two-body dimer calculation. The energies, gradients, and Hessian elements were all extrapolated to the complete basis set (CBS) limit using a two-point TQ extrapolation of both the Hartree–Fock^78^ and correlation energy contributions.^79^ Energies and gradients at the CCSD(T)/CBS limit were estimated by correcting the MP2/CBS limit values with the difference between CCSD(T) and MP2, ΔCCSD(T) ≈ CCSD(T) – MP2, computed in the aug-cc-pVDZ basis set. MP2 phonons were used to evaluate *F*_vib_ in the CCSD(T) calculations. The MM contributions in eqn (1) were computed using the Amoeba force field and Tinker 6.3.^80^ Intermolecular force field parameters for CO_2_ were generated using Poltype version 1.1.3.^81^

Once the crystal structures were obtained as a function of temperature and pressure, various thermodynamic properties were computed using standard expressions from statistical mechanics. Ideal gas behavior was assumed for the vapor phase. Additional methodological details are provided in the ESI.†

The relative rigidity and lack of many-body polarization effects makes carbon dioxide a good candidate for simple, fixed charge force field models, though the importance of many-body dispersion effects has been noted.^82^ For comparison with the electronic structure results, the predictions here were repeated using the empirical CO_2_ potential of Cygan and co-workers.^83^ This flexible, three-point model includes standard harmonic stretch and bend terms, point-charge electrostatics, and Lennard-Jones dispersion/repulsion terms. It was particularly parameterized to reproduce vibrational spectra, which should help it capture the phonon contributions. The carbon dioxide quadrupole moment also proves important for modeling its solid state,^84^ and the point charges in this force field generate a molecular quadrupole of –4.22 D Å, in good agreement with the experimental value of –4.27 ± 0.18 D Å.^85^ Additional test calculations with the TraPPE force field,^86^ which uses the same functional form but slightly different empirical parameters, produced similar results (not presented here). Of course, many other CO_2_ potentials exist, and a more elaborate or physical potential (*e.g.*ref. 87) might perform better than the particular one chosen here.

## 3 Results and discussion

The next sections compare the predicted and experimental values for thermal expansion, thermodynamic properties, and the bulk modulus. All predicted values plotted in figures here are tabulated in the ESI.†

### 3.1 Thermal expansion

**Fig. 1:**
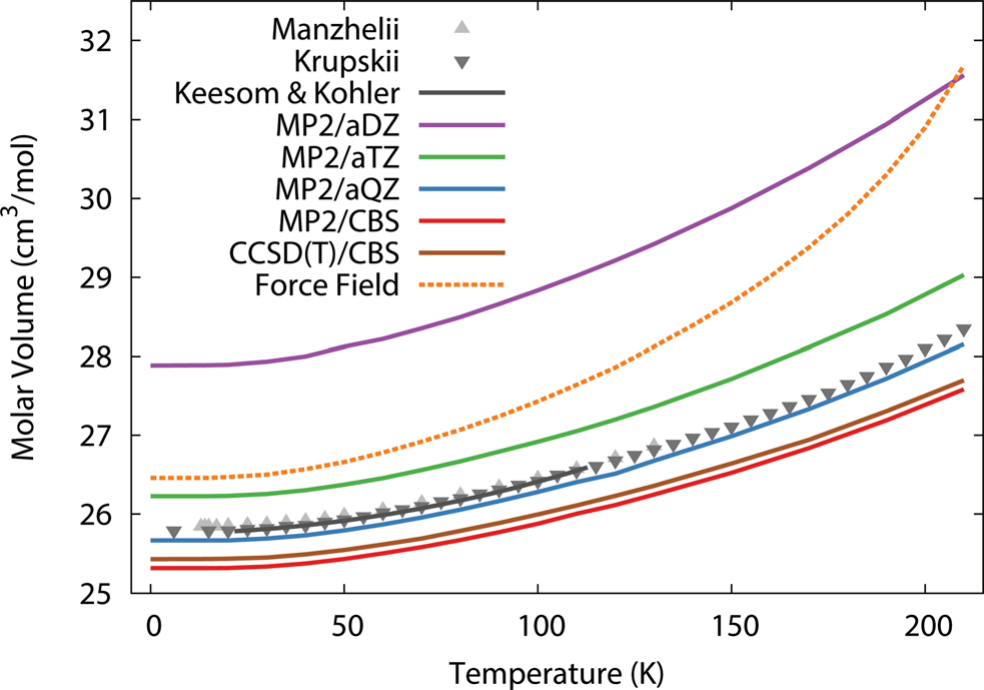
Predicted thermal expansion of the CO_2_(s) unit cell compared to the experimental values^88^–^91^ in gray.

To begin, we predict the thermal expansion of the CO_2_ lattice at atmospheric pressure by optimizing the quasiharmonic Gibbs free energy at a series of different temperatures. At 1 atm, the *PV* term only contributes ∼0.01 kJ mol^–1^ to the overall energy, so it was neglected here. Fig. 1 compares these predictions against experimental results from Manzhelii *et al.*,^88^ Krupskii *et al.*,^89^ and the low-temperature fit (20–114 K) of Keesom and Köhler.^90^,^91^ In a small aug-cc-pVDZ basis set, MP2 substantially underbinds the crystal, leading to a substantial over-estimation of the unit cell volume. As we approach the complete-basis-set (CBS) limit, however the MP2 prediction improves dramatically, with MP2/CBS underestimating the cell volume by only 2–3%. Fortuitously, the slightly smaller aug-cc-pVQZ basis performs even better, with predicted volumes lying within ∼0.5% of experiment.

The errors are nearly constant across the entire temperature range. For instance, MP2/CBS underestimates the volume by 0.5 cm^3^ mol^–1^ (2%) at low temperatures, and this error increases to only 0.7 cm^3^ mol^–1^ (3%) at the sublimation point (194.7 K). Most of the error is present already in the lowest temperature results, which suggests it largely stems from the underlying fragment-based electronic structure treatment, rather than from the quasiharmonic approximation. The treatment of phonon dispersion *via* lattice dynamics is also important here. Using *Γ*-point frequencies only causes the model to underestimate the rate of thermal expansion noticeably (see ESI†).

One might hope to obtain further improvements by moving beyond second-order perturbation theory to the CCSD(T) level. However, previous work indicates that correlation energy contributions beyond second-order perturbation theory are small in crystalline CO_2_, with the lattice energy shifting by only ∼0.3 kJ mol^–1^ between MP2 and CCSD(T).^41^ Here, refining the thermal expansion predictions at the CCSD(T)/CBS level (with the free energy computed as the sum of CCSD(T) internal energies and MP2 vibrational free energy contributions) reduces the errors by only 0.1 cm^3^ mol^–1^. Nevertheless, these results show that large-basis electronic structure calculations plus the quasiharmonic approximation model the temperature dependence of the carbon dioxide unit cell volume very reliably all the way up to the sublimation point.

For comparison, the force field potential performs quite well at low temperature, predicting a cell volume that is roughly on par with the MP2/aug-cc-pVTZ calculation with orders of magnitude lower computational cost. However, as the temperature increases, the force field model expands the crystal volume much too rapidly.

### 3.2 Thermodynamic properties

Given the excellent treatment of thermal expansion, we next investigate the model's ability to predict thermodynamic properties such as the heat capacity and the enthalpies and entropies of sublimation. Such properties are critical to determining polymorph stability at finite temperatures. For each of these properties, predictions were made with and without the thermal expansion provided by the quasiharmonic approximation.

**Fig. 2:**
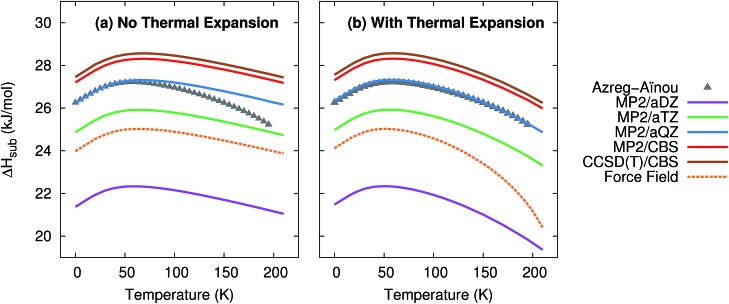
Predicted enthalpies of sublimation at 1 atm (a) neglecting thermal expansion and (b) with quasiharmonic thermal expansion, relative to the empirical data of Azreg-Aïnou.^92^

Fig. 2 plots the enthalpy of sublimation at 1 atm relative to the experimentally-derived Δ*H*_sub_ determined by Azreg-Aïnou.^92^ Azreg-Aïnou derived these values using fits to the experimentally observed heat capacity and vapor pressure data, ideal gas partition functions, various small corrections for gas imperfection, and other details.

The sublimation enthalpy is dominated by the crystal lattice energy. The zero-point and thermal enthalpy corrections account for only ∼10% (at low temperature) to ∼25% or more (at the sublimation point) of the total sublimation enthalpy. Accordingly, the sublimation enthalpy should behave similarly to the lattice energy with regard to the basis set: small-basis MP2 underestimates the CO_2_ lattice energy significantly, but using large basis sets mostly corrects this error.^40^,^41^ As expected, small basis sets predict a sublimation enthalpy that is too small, while MP2/aug-cc-pVQZ fortuitously predicts a sublimation enthalpy in almost perfect agreement with experiment. Extrapolating to the complete-basis-set limit produces a sublimation enthalpy that overestimates the experimental value by only 1.0–1.1 kJ mol^–1^. CCSD(T)/CBS binds crystalline CO_2_ slightly more,^41^ which increases the sublimation enthalpy further, to a value 1.3–1.4 kJ mol^–1^ too large. This accuracy is near the limit of what is achievable with modern electronic structure theory. Errors in the lattice energy of 1–2 kJ mol^–1^ represent a best-case scenario for practical molecular crystal calculations,^39^,^41^ while errors of several kJ mol^–1^ are more typical.^25^,^26^,^30^,^31^,^46^

Fig. 2 also highlights how the approximate treatment of anharmonicity and thermal expansion *via* the quasiharmonic approximation proves essential to capturing the proper temperature dependence above 50 K. Without the quasiharmonic approximation, the theoretical calculations substantially overestimate the sublimation enthalpy at higher temperatures. When the quasiharmonic approximation is employed, however, the calculations obtain the correct curvature across a 200 K temperature range. Both the MP2/CBS and CCSD(T)/CBS results predict the maximum in the sublimation enthalpy at 59 K, in excellent agreement with the 58.829 K reported by Azreg-Aïnou.^92^

Once again, the force field model used here performs almost as well as the MP2/aug-cc-pVTZ results at low temperatures or when thermal expansion is neglected. However, the exaggerated thermal expansion seen in Fig. 1 is reflected in poor prediction of the sublimation enthalpy at warmer temperatures.

**Fig. 3:**
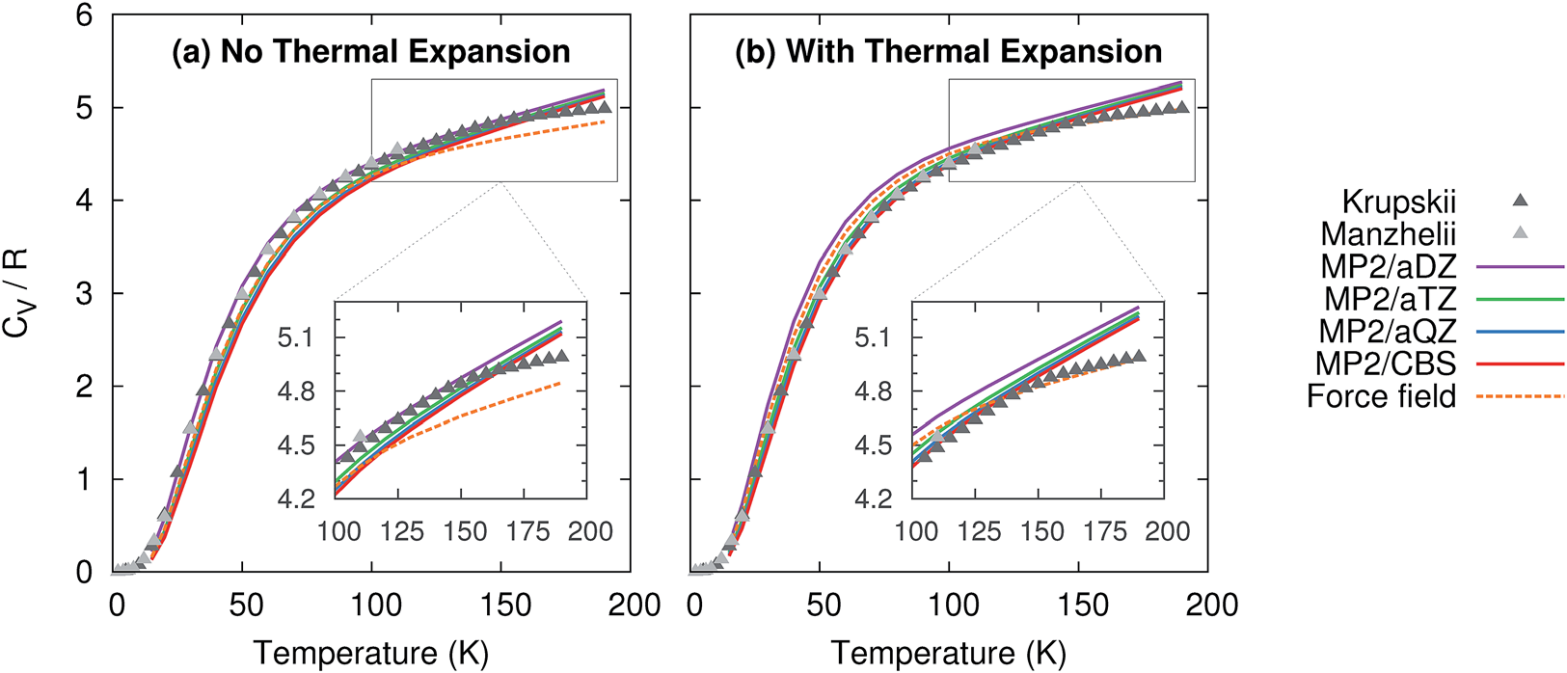
Predicted isochoric heat capacity (a) neglecting thermal expansion and (b) with quasiharmonic thermal expansion relative to the experimental data of Krupskii *et al.*^89^ and Manzhelii *et al.*^88^

Given the high accuracy of the MP2 and CCSD(T) sublimation enthalpy predictions as a function of temperature, it is not surprising that the isochoric heat capacity, *C*_V_, is also predicted reliably (Fig. 3). Note that CCSD(T) results are not provided because CCSD(T) phonons are unavailable.† For the heat capacity, all models perform fairly well. Neglecting thermal expansion fortuitously causes small aug-cc-pVDZ basis MP2 to out-performs what should be the more accurate large basis calculations relative to the experimental values of Krupskii *et al.*^89^ and Manzhelii *et al.*^88^ When thermal expansion is included, on the other hand, the accuracy of the predictions does improve with increasing basis set, as one generally expects.

Similar to previously published small-basis MP2 results,^35^ we find that MP2 underestimates the heat capacity slightly at low temperature. Errors of 1–1.5 J mol^–1^ K^–1^ (0.1–0.2*R*) are observed below 50 K. However, the results here perform better than the earlier MP2 ones at moderate temperatures (*e.g.* ∼50–150 K), with errors typically well below 1 J mol^–1^ K^–1^ (0.1*R*) in the range 50–150 K. At higher temperatures, the predictions begin to deviate more noticeably from the experimental data, probably due to increased anharmonicity in the phonons. This suggests that one might expect larger deviations from the correct temperature-dependence of the sublimation enthalpy at higher temperatures. Nevertheless, on the whole, MP2 predicts the heat capacity accurately across a fairly wide temperature range.

For comparison, the force field model behaves similarly to MP2/aug-cc-pVDZ and aug-cc-pVTZ at low and intermediate temperatures, but it asymptotes more quickly than the MP2 heat capacities at higher temperatures. This actually leads to a slightly better prediction of the heat capacity near 200 K when thermal expansion is included. Of course, this result is somewhat fortuitous, given the problems seen earlier in the volume and sublimation enthalpy.

Entropy also plays a critical role in phase stability. The entropy of sublimation at the sublimation point (*T* = 194.7 K) is well-known,^93^ but we are not aware of any existing tabulation of the experimental sublimation entropy as a function of temperature. Accordingly, we derived an empirical sublimation entropy from existing experimental data according to:
2

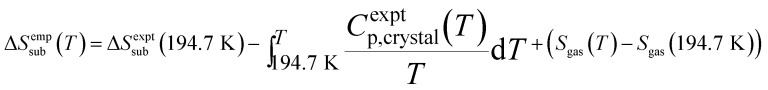




This expression relates the sublimation entropy at a given temperature to the experimental value at 194.7 K plus corrections for how the entropies of the crystal and the gas change as a function of temperature. The changes in the entropy of the crystal were computed *via* integration of the experimental isobaric heat capacities,^93^ while the gas contributions were evaluated using ideal gas partition functions and the experimentally determined rotational constant^94^ and vibrational frequencies.^95^ See the ESI† for details.

**Fig. 4:**
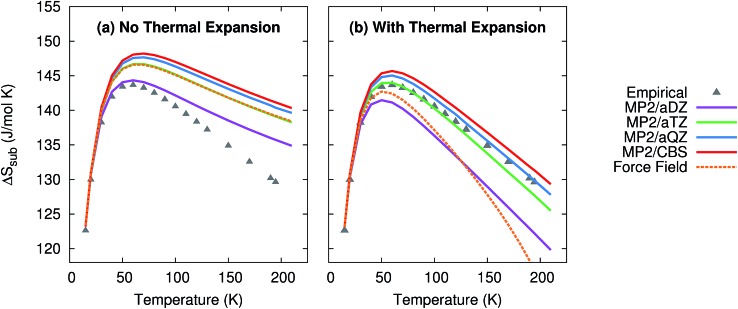
Predicted entropies of sublimation at 1 atm (a) neglecting thermal expansion and (b) with quasiharmonic thermal expansion, relative to the data empirically derived from experiment.

As shown in Fig. 4, the quasiharmonic treatment of thermal expansion proves critical to obtaining the correct temperature dependence of the entropy. Without thermal expansion, MP2/CBS overestimates the sublimation entropy above 50 K by up to 9%. In contrast, including thermal expansion dramatically reduces the errors, predicting the sublimation entropy to within 1–2% throughout the 200 K temperature range. For comparison, without thermal expansion, the force field mimics MP2/aug-cc-pVTZ. However, once thermal expansion is included, the force field predicts an entropy of sublimation that decreases much too rapidly at higher temperatures.

Finally, the sublimation point can be predicted by combining the enthalpy and entropy of sublimation to determine the temperature where Δ*G*_sub_ = 0. As shown in Table 1, small aug-cc-pVDZ basis MP2 calculations underestimate the sublimation temperature by 30 K. Increasing the basis set, however, allows one to predict the experimental sublimation temperature of 194.7 K within 5 K (MP2/CBS) or 6 K (CCSD(T)/CBS) when thermal expansion is included. The CCSD(T) enthalpy and entropy of sublimation at 194.7 K are predicted to within 1.4 kJ mol^–1^ (6%) and 1.9 J mol^–1^ K^–1^ (1%), respectively.

**Table 1:** Predicted sublimation temperatures *T*_sub_ at 1 atm, and the corresponding enthalpies and entropies of sublimation at the experimental sublimation point of 194.7 K

	No thermal expansion			With thermal expansion		
	*T*_sub_ (K)	Δ*H*_sub_ (194.7 K) (kJ mol^–1^)	Δ*S*_sub_ (194.7 K) (J mol^–1^ K^–1^)	*T*_sub_ (K)	Δ*H*_sub_ (194.7 K) (kJ mol^–1^)	Δ*S*_sub_ (194.7 K) (J mol^–1^ K^–1^)
Force field	172.9	24.0	139.2	183.4	21.5	116.9
MP2/aug-cc-pVDZ	157.1	21.2	135.7	163.6	19.8	122.2
MP2/aug-cc-pVTZ	178.9	24.9	139.3	185.3	23.7	127.8
MP2/aug-cc-pVQZ	187.1	26.3	140.6	193.4	25.3	130.0
MP2/CBS	193.2	27.3	141.4	199.2	26.0	131.5
CCSD(T)/CBS^*a*^	194.9	27.6	^*b*^	201.0	26.6	^*b*^
Giauque and Egan^93^				194.7	25.2	129.6

^*a*^Using MP2/CBS frequencies and thermal contributions.

^*b*^Identical to the MP2/CBS value.

If one neglects thermal expansion, CCSD(T)/CBS predicts a sublimation temperature of 194.9 K, which agrees almost perfectly with the experimental temperature. However, this accuracy results from fortuitous error cancellation—the Δ*H*_sub_ and Δ*S*_sub_ values at 194.7 K are both 9–10% too large. The force field predicts sublimation temperature of 172.9 K without thermal expansion, or 183.4 K with thermal expansion. As before, these values are similar to those obtained from MP2/aug-cc-pVTZ. One should note, however, that in the case where thermal expansion is included, the force field enthalpy and entropy of sublimation are both underestimated considerably to produce the relatively good estimate for the sublimation temperature.

Once again, these sublimation point predictions reiterate the importance of modeling thermal expansion. More importantly, they hint toward a future where high-quality *ab initio* prediction of phase diagrams as a function of both temperature and pressure may be routine.

### 3.3 Bulk modulus

Mechanical properties like the bulk modulus are also of considerable interest for many applications. To obtain the bulk modulus, one typically measures the crystal volume as a function of pressure, and then fits the resulting data to an equation of state, treating the isothermal bulk modulus at zero pressure (*B*_0_), its first pressure derivative (*B*′_0_), and the unit cell volume at zero pressure (*V*_0_) as adjustable parameters. Many equations of state exist, including the third-order Birch–Murnaghan^96^ and Vinet^97^ equations. Non-linear least squares fits to these equations of state can be problematic, with the resulting fit parameters being ill-constrained (*i.e.* a wide range of parameters produce comparably good fits) and highly correlated.^98^,^99^ The resulting parameters depend strongly on the reference volume at zero pressure (*V*_0_), especially when using the Birch–Murnaghan equation of state.^98^ This challenge is particularly acute at room temperature, where crystalline carbon dioxide does not exist at zero pressure, and *V*_0_ must be obtained *via* extrapolation from finite-pressure volumes. Hence, considerable uncertainty surrounds the experimental bulk modulus parameters for CO_2_.^88^,^89^,^98^–^104^

Theory can predict the pressure–volume data at a given temperature to fit the equation of state, and it can predict the zero-pressure unit cell volume *V*_0_*via* direct geometry optimization. This latter feature enables one to validate the *V*_0_ obtained in a fit or even constrain *V*_0_, if necessary, in order to extract *B*_0_ and *B*′_0_. Previous theoretical studies have predicted a variety of bulk modulus values,^35^,^59^,^60^,^62^ though the difficulty in computing these parameters reliably has been noted.^35^ These earlier studies either neglected thermal expansion^35^,^59^,^62^ or omitted van der Waals dispersion,^59^,^60^ which is significant for CO_2_.^49^,^62^ Here, we demonstrate that the combination of high-level electronic structure calculations and a quasiharmonic treatment predicts *B*_0_ and *B*′_0_ in excellent agreement with the best experimental values across a wide range of temperatures.

**Fig. 5:**
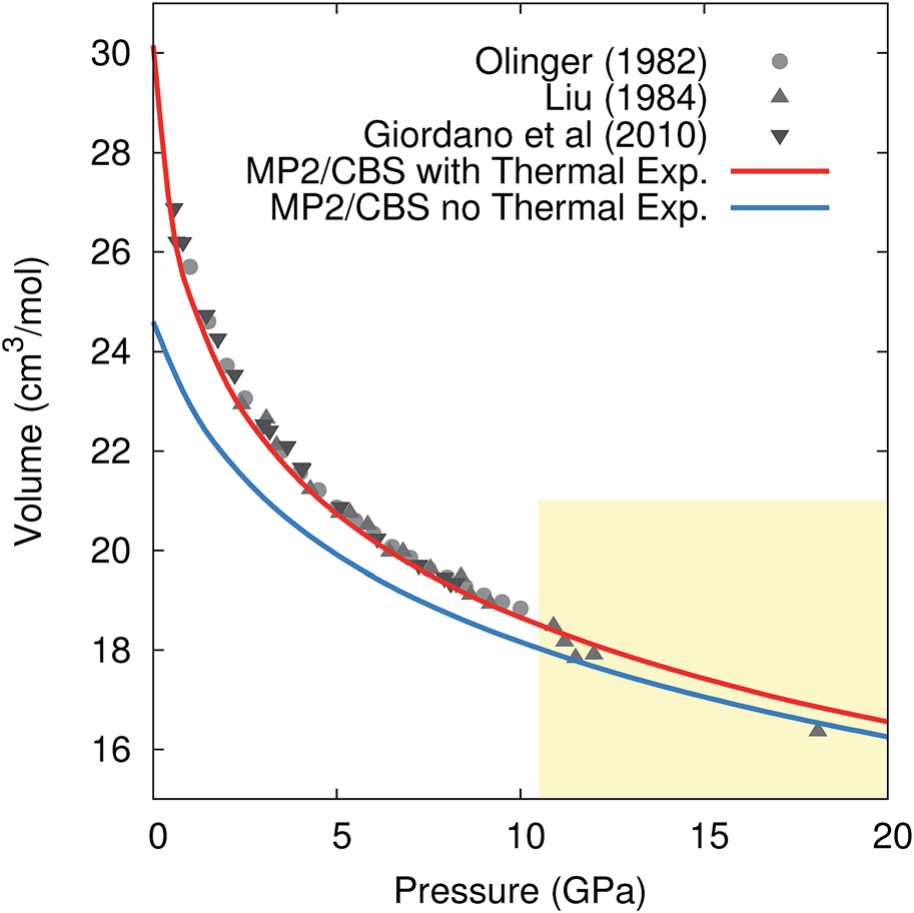
Comparison of the experimental and predicted MP2/CBS pressure *versus* volume curves at 296 K, with and without quasiharmonic thermal expansion. Note that the drop in the experimental volumes above 10 GPa (shaded region) is believed to reflect a transition to phase III,^105^ while the calculations presented are for phase I throughout.

Pressure *versus* volume curves were calculated by optimizing the crystal geometry under a series of external pressures ranging from 0–10 GPa (0–20 GPa for 296 K) at 0 K, 130 K, 190 K, and 296 K under the quasiharmonic approximation. Analogous calculations were also performed at 0 K without the quasiharmonic vibrational contribution *F*_vib_. As a representative example, Fig. 5 compares the experimental and room-temperature MP2/CBS predicted pressure *versus* volume curves with and without the inclusion of quasiharmonic thermal expansion. Inclusion of thermal expansion proves critical to reproducing the experimental pressure/volume data. Differences between the curves with and without thermal expansion persist even at 20 GPa, where one might have hoped that the high external pressure would obviate the need to treat thermal expansion.

For each temperature and level of theory, the values of *V*_0_, *B*_0_, and *B*′_0_ were extracted *via* non-linear least squares fitting to the Vinet equation of state,
3

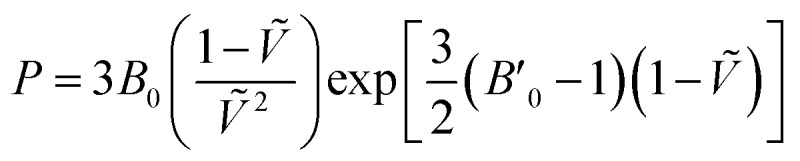

where = (*V*/*V*_0_)^1/3^. The Vinet equation of state fits prove much more robust than the Birch–Murnaghan ones for the CO_2_*P*–*V* curves. The fits to the predicted *P*–*V* curves were validated by performing a second set of fits in which *V*_0_ was fixed at the molar volume obtained directly by optimizing the crystal at a given temperature and zero pressure. Both sets of fits produced very similar volumes and bulk moduli. See ESI† for details.

**Fig. 6:**
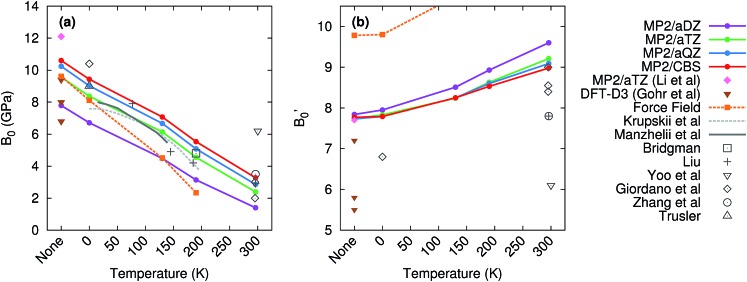
Experimental (gray) and predicted (colored) values of the (a) bulk modulus *B*_0_ and (b) its first pressure derivative *B*′_0_. The label ‘‘none’’ in the figures refers to calculations which neglect temperature and the quasiharmonic approximation entirely.

Fig. 6 compares the predicted values of *B*_0_ and *B*′_0_ obtained here to previously reported theoretical and experimental values. Without the quasiharmonic approximation, the bulk modulus parameters obtained here are similar to earlier predictions using MP2/aug-cc-pVTZ by Li and co-workers^35^ and various dispersion-corrected density functional calculations.^62^ However, the bulk modulus shrinks several-fold upon heating to room temperature, and the treatment of thermal expansion provided by the quasiharmonic approximation is required to capture that.

Basis set effects are also fairly important for the bulk modulus—the MP2 *B*_0_ value increases by 30–130% (depending on temperature) from a small aug-cc-pVDZ basis to the complete basis set limit. The pressure derivative *B*′_0_ is less sensitive to basis set. Correlation beyond second-order perturbation theory proves relatively unimportant here. At 190 K, switching from MP2 to CCSD(T) increases *V*_0_ by 0.1 cm^3^ mol^–1^, increases *B*_0_ by 0.2 GPa, and does not alter *B*′_0_ (see Table S2 in the ESI†).

The experimental bulk modulus data exhibits considerable scatter, but the bulk moduli *B*_0_ predicted here are consistent with most of the literature data across all temperatures (Fig. 6). Less experimental data exists for the first-pressure derivative *B*′_0_, but values predicted here are in good agreement with the available experimental ones. MP2/CBS overestimates the reported room temperature experimental values of *B*′_0_ by 5–15%, but the predicted value lies within the typical experimental error bars. For instance, the MP2/CBS predictions of *B*_0_ = 3.3 GPa and *B*′_0_ = 9.0 at 296 K are in excellent agreement with the Vinet equation of state fit by Giordano *et al.*,^99^ which found *B*_0_ = 3 ± 1 GPa and *B*′_0_ = 8.4 ± 0.8. The MP2 predictions are also consistent with the Vinet fits to the Olinger^101^ and Liu^98^ experimental *P*–*V* curves reported by Giordano *et al.*,^99^ which exhibit even larger uncertainties. Moreover, the MP2 predictions compare well with experimental bulk modulus values at other temperatures, including those from Krupskii *et al.*,^89^ Manzhelii *et al.*,^88^ Bridgman,^100^ Liu,^98^ and Trusler.^103^

The experimentally obtained *B*_0_ = 6.2 GPa and *B*′_0_ = 6.1 values at 300 K reported by Yoo *et al.*^102^ are considerable outliers with respect to both our theoretical predictions and the other experimental values. Ref. 102 provides few details of the data or fitting procedure used for phase I, but their reported zero-pressure volume *V*_0_ = 25.1 cm^3^ mol^–1^ is substantially smaller than the values of ∼30 ± 2 cm^3^ mol^–1^ found by Giordano *et al.*,^99^ 31.4 cm^3^ mol^–1^ inferred by Liu,^98^ and 30.1 cm^3^ mol^–1^ predicted by MP2/CBS geometry optimization. In fact, their room-temperature *V*_0_ is smaller than the experimental volume of 25.8 cm^3^ mol^–1^ at 6 K.^89^ Therefore, these bulk modulus values probably reflect a spurious fit to the experimental data.

For comparison, the force field predicts a reasonable bulk modulus without temperature or at 0 K (where only zero-point effects are included), but it exaggerates the thermal expansion and predicts that the bulk modulus decreases much more rapidly with temperature than experiments or the MP2 calculations indicate. Similarly, the first pressure derivative of the bulk modulus is overestimated and increases too quickly with temperature in the force field model. Note too that at 296 K, the CO_2_ crystal proved unbound with the force field model, and no reasonable fit could be found to the Vinet equation of state.

In the end, the electronic structure results here demonstrate that theory can provide a powerful tool for predicting properties such as the bulk modulus, which can be difficult to extract reliably from experiment. The calculations here provide support for the room-temperature bulk moduli obtained by Giordano *et al.* and others, while simultaneously suggesting that some reported values are unlikely. Furthermore, theory can be used to identify a plausible experimental zero-pressure volume, which is often a key step in extracting bulk modulus parameters from experiment. Finally, the treatment of thermal expansion proves critical to predicting the correct the temperature-dependence of the bulk modulus parameters.

## 4 Conclusions

In summary, we are rapidly transitioning into an era where electronic structure theory can directly predict a wide range of experimentally observable molecular crystal properties under practical temperature and pressure conditions. As shown here, the combination of accurate electronic structure theory calculations and a quasiharmonic treatment of thermal expansion enables one to predict crystal structures, thermodynamics, and mechanical properties for phase I carbon dioxide in excellent agreement with experiment. While the simple force field considered here behaves very well at low temperatures and predicts results on roughly par with those from MP2/aug-cc-pVTZ, the electronic structure calculations provide substantially improved agreement with experiment at higher temperatures.

The performance of the quasiharmonic approximation seen here does start to degrade at higher temperatures, so it remains to be seen how well it performs in larger crystals which are stable at room temperature and above. Still, the excellent performance seen here up to 200 K (or room temperature for the bulk modulus) for carbon dioxide provides considerable cause for optimism. Of course, the increased anharmonicity found in larger, more flexible organic molecules will also create new challenges for the simple quasiharmonic approximation used here.

The quantum mechanical calculations here are made feasible by fragment-based electronic structure methods, which make MP2 and even coupled cluster calculations computationally affordable for molecular crystals. Although the extrapolated complete-basis MP2 and CCSD(T) calculations employed on CO_2_ here would be much more computationally challenging for a pharmaceutical crystal, in many cases one can probably obtain useful predictions using a lower level of theory. MP2/aug-cc-pVTZ already predicts many of the properties in reasonable agreement with experiment, albeit with several-fold less computational effort than the larger-basis results. It may provide a useful level of theory for modeling crystals of larger molecules. Continuing algorithmic developments and decreasing costs of computer hardware will hopefully make finite-temperature predictions on chemically interesting organic molecular crystals routine in the near future.

## Supplementary Material

Supplementary information
