# Supplementary material for: Predicting finite-temperature properties of crystalline carbon dioxide from first principles with quantitative accuracy
Source: Chem Sci. 2015 Sep 29;7(1):246–55. doi: 10.1039/c5sc03014e (PMC5952317; doi:10.1039/c5sc03014e)
Supplement: Supplementary file 1 [file SC-007-C5SC03014E-s001.pdf]

Electronic Supplementary Material (ESI) for Chemical Science.
This journal is © The Royal Society of Chemistry 2015

Supporting Information for "Predicting finite-temperature properties of crystalline carbon dioxide from first principles with quantitative accuracy"

by Y. Heit, K. Nanda, and G. Beran*

Contents

1 Method for computing the thermal expansion **2**

2 Method for computing thermodynamic parameters **3**

3 Procedure for obtaining empirical entropy data **4**

4 Equation of state fitting procedures **6**

5 Importance of phonon dispersion **9**

6 Intermolecular Amoeba force field parameters for CO_2 **10**

7 Tables of predicted properties **10**
7.1 Temperature-dependence of the crystal structure 10
7.2 Isochoric heat capacities 12
7.3 Enthalpies of sublimation 13
7.4 Entropies of sublimation 15
7.5 Bulk moduli 17

Note: The short-hand "aXZ" is used here to refer to the aug-cc-pVXZ basis (where X = D, T, or Q).

*Department of Chemistry, University of California, Riverside, California, 92521, USA. Email: gregory.beran@ucr.edu

1 Method for computing the thermal expansion

To determine the structure of phase I carbon dioxide at a given temperature T and pressure P, one minimizes the Gibbs free energy G with respect to both the atomic positions in the unit cell and the unit cell parameters (a, b, c, α, β, γ).

$$G(T,P) = U_{el} + PV + F_{vib}(T) \tag{1}$$

where U_{el} is the internal electronic energy, PV is the pressure-volume contribution, and F_{vib} represents the Helmholtz vibrational free energy contribution. This latter contribution is expressed in terms of the harmonic phonons $\omega_{k,i}$ evaluated at multiple **k**-points in reciprocal space:

$$F_{vib}(T) = \frac{N_A}{n}\sum_k \sum_i \left(\frac{\hbar\omega_{k,i}}{2} + k_b T \ln\left[1 - \exp\left(-\frac{\hbar\omega_{k,i}}{k_b T}\right)\right]\right) \tag{2}$$

where n is the number of unit cells in the supercell approximation and N_A is Avogadro's number.

Both U_{el} and F_{vib} are computed using the fragment-based hybrid many-body interaction (HMBI) model,[1–4] which allows one to perform high-level MP2 and even coupled cluster calculations on periodic systems like molecular crystals with reasonable computational cost. HMBI decomposes the intermolecular interactions in a crystal according to a many-body expansion,

$$U_{El} = E^{QM}_{1-body} + E^{QM}_{SR\ 2-body} + E^{MM}_{LR\ 2-body} + E^{MM}_{many-body} \tag{3}$$

The important intramolecular (1-body) and short-range pairwise (SR 2-body) interactions are treated with quantum mechanics (QM), while the generally weaker long-range pairwise (LR 2-body) and many-body contributions are approximated with a polarizable molecular mechanics (MM) force field. In practice, the short-range 2-body QM treatment includes interactions involving molecules in the unit cell and those in nearby periodic image cells, while the MM terms are used to capture the long-range periodicity of the crystal via Ewald summation.

The phonons at a given **k**-point are evaluated by constructing and then diagonalizing the mass-weighted dynamical matrix using the supercell approach,

$$D_{\alpha,\beta}\left(l,l';\mathbf{k}\right) = \frac{1}{\sqrt{M_l M_{l'}}} \sum_{\kappa} \frac{\partial^2 U_{El}}{\partial\alpha_{l(0)}\partial\beta_{l'(\kappa)}} \exp\left(-2\pi i \mathbf{k}\cdot\delta\mathbf{x}(0,\kappa)\right) \tag{4}$$

where $\frac{\partial^2 U_{El}}{\partial\alpha_{l(0)}\partial\beta_{l'(\kappa)}}$ are individual elements in the supercell Hessian[5,6] involving the α-coordinate of atom l in the central unit cell (index 0) and the β-coordinate of atom l' in the unit cell with index κ, and $\delta\mathbf{x}(0,\kappa)$ is the displacement vector between these two atoms. The M's are the atomic masses. The exponential introduces the phase shift in the harmonic motions of the periodic image atoms relative to the atoms in the central unit cell.

Unlike conventional periodic boundary condition models, the fragment approach used in HMBI enables these equations to be evaluated for the supercell Hessian with minimal additional effort beyond the normal unit cell Hessian.[6,7] All the necessary supercell QM contributions to the force constants in Eq 4 can be constructed from contributions already available from the standard unit cell Hessian. The only new contribution required is the full supercell Hessian at the MM level. This allows one to capture phonon dispersion by using a large supercell and sampling many **k** points with comparatively low additional computation cost.

The treatment of F_{vib} is potentially very computational demanding, since it would normally require many cycles of geometry optimization and a phonon calculation for each update in the unit cell parameters. Instead, we approximate the phonons for a given crystal volume using the quasiharmonic approximation (QHA). The QHA relates the i-th phonon frequency ω_i at a given volume V to a reference

frequency $\omega_{i,ref}$ obtained at some reference volume V_{ref} via the Grüneisen parameter for that phonon mode γ_i,

$$\omega_i = \omega_{i,ref} \left(\frac{V}{V_{ref}} \right)^{-\gamma_i} \tag{5}$$

where

$$\gamma_i = -\left(\frac{\partial \ln \omega_i}{\partial \ln V} \right) \tag{6}$$

Overall, then, a reference crystal structure, unit cell volume, and phonon frequencies are obtained at zero temperature and pressure by minimizing U_{el}, and the harmonic phonon modes are computed using lattice dynamics. Two additional rounds of geometry optimization and phonon calculation are performed with fixed unit cell parameters at cell volumes which are slightly larger and smaller than V_{ref}. Here, the cell volume was expanded/contracted by 10 Å^3, or roughly 5%. The Grüneisen parameter for each of the 3N vibrational modes is computed via finite difference using a linear model.[8] The phonon frequencies at the different volumes were matched via maximum overlap of the phonon eigenmodes.

With the reference frequencies, reference volume, and Grüneisen parameters obtained from these three optimizations and lattice dynamics phonon calculations, one can evaluate F_{vib} at any given temperature and unit cell volume. That allows one to find the crystal structure at any temperature and pressure by minimizing the Gibbs free energy (Eq 1). Other properties like the heat capacity, enthalpy, and entropy can be calculated via standard partition function expressions based on the optimized structures and quasiharmonic phonon frequencies.

As described in the main paper, the QM contributions in the HMBI model were computed with MP2 or CCSD(T). At the MP2 level, analytic gradients were used, while the Hessian elements were calculated via finite difference of the gradients. The lattice dynamics phonon were performed on a $3 \times 3 \times 3$ supercell using a $3 \times 3 \times 3$ Monkhorst-Pack grid of **k** points. At the CCSD(T) level, analytic gradients are computationally expensive and are not widely available. Accordingly, the gradients were evaluated via finite difference of the energies. However, computing CCSD(T) second nuclear derivatives via finite difference of the energies introduced too much numerical noise into the two-body Hessian contributions, which made it difficult to obtain useful phonon frequencies. Therefore, for the CCSD(T) calculations, F_{vib} was evaluated using MP2/CBS frequencies and Grüneisen parameters.

2 Method for computing thermodynamic parameters

Using the crystal structures optimized at a given temperature according to Section 1 and the quasi-harmonic estimates for the phonon frequencies, one can compute the various thermodynamic properties reported in the paper as follows:

Enthalpy of sublimation: The sublimation enthalpy is given as the difference between the enthalpy of the gas and 1/4 the enthalpy of the crystal (which has 4 molecules in the unit cell).

$$\Delta H_{sub} = H_{gas} - \frac{1}{4} H_{crystal} \tag{7}$$

The enthalpy of the crystal is computed as the sum of the electronic energy of the crystal $U_{el,crystal}$ plus a pressure-volume term PV and a vibrational internal energy contribution $E_{vib,crystal}$:

$$H_{crystal} = U_{el,crystal} + PV + E_{vib,crystal} \tag{8}$$

where the vibrational internal energy contribution is given as:

$$E_{vib,crystal} = \frac{N_A}{n} \sum_{k}^{n} \sum_{i} \left(\frac{\hbar\omega_{k,i}}{2} + \frac{\hbar\omega_{k,i}}{exp\left(\frac{\hbar\omega_{k,i}}{k_b T}\right) - 1} \right) \tag{9}$$

The gas phase was modeled using standard ideal gas partition functions. Of course, the gas may exhibit deviations from ideality at the low temperatures considered here, but they are hopefully not too large. The enthalpy of the gas is given as the sum of electronic ($U_{el,gas}$), translational ($3/2RT$), rotational (RT), vibrational (E_{vib}) terms plus an additional factor of RT from the PV term.

$$H_{gas} = U_{el,gas} + \frac{3}{2}RT + RT + E_{vib} + RT \tag{10}$$

where E_{vib} is defined as:

$$E_{vib,gas} = N_A \sum_{i}^{4} \left(\frac{\hbar\omega_i}{2} + \frac{\hbar\omega_i}{exp\left(\frac{\hbar\omega_i}{k_b T}\right) - 1} \right) \tag{11}$$

Entropy of sublimation: The entropy of sublimation was computed as,

$$\Delta S_{sub} = S_{gas} - \frac{1}{4} S_{vib,crystal} \tag{12}$$

Once again, the gas was modeled using standard ideal gas partition function contributions from translation, rotation, and vibration:

$$S_{gas} = S_{trans,gas} + S_{rot,gas} + S_{vib,gas} \tag{13}$$

where the terms are defined as:

$$S_{trans,gas} = R \ln \left[\left(\frac{2\pi m k_b T}{h^2} \right)^{3/2} \left(\frac{k_b T e^{5/2}}{p} \right) \right] \tag{14}$$

$$S_{rot,gas} = R \ln \left(\frac{8\pi^2 e T I k_b}{\sigma h^2} \right) \tag{15}$$

$$S_{vib,gas} = N_A \sum_{i}^{4} \left(\frac{\hbar\omega_i}{T\left(exp\left(\frac{\hbar\omega_i}{k_b T}\right) - 1\right)} - k_b \ln \left[1 - exp\left(-\frac{\hbar\omega_i}{k_b T} \right) \right] \right) \tag{16}$$

Note that the symmetry factor σ in S_{rot} is 2 for carbon dioxide.

$$S_{vib,crystal} = \frac{N_A}{n} \sum_{k}^{n} \sum_{i} \left(\frac{\hbar\omega_{k,i}}{T\left(exp\left(\frac{\hbar\omega_{k,i}}{k_b T}\right) - 1\right)} - k_b \ln \left[1 - exp\left(-\frac{\hbar\omega_{k,i}}{k_b T} \right) \right] \right) \tag{17}$$

Heat capacity: The isochoric heat capacity of the crystal was computed using the standard harmonic oscillator expression,

$$C_v = \frac{R}{n} \sum_{k}^{n} \sum_{i} \left[\left(\frac{\hbar\omega_{k,i}}{k_b T\left(exp\left(\frac{\hbar\omega_{k,i}}{k_b T}\right) - 1\right)} \right)^2 exp\left(\frac{\hbar\omega_{k,i}}{k_b T} \right) \right] \tag{18}$$

3 Procedure for obtaining empirical entropy data

The empirical entropies were derived from experimental data according to:

$$\Delta S_{sub}^{emp}(T) = \Delta S_{sub}^{expt}(194.7K) - \int_{194.7K}^{T} \frac{C_{p,crystal}^{expt}(T)}{T} dT + (S_{gas}(T) - S_{gas}(194.7K)) \tag{19}$$

This expression relates the sublimation entropy at a given temperature to the experimental sublimation entropy at the sublimation point (i.e. ΔS_{sub}(194.7 K) = 129.62 J/mol K), as reported by Giauque and Egan.[9] By construction, Eq 19 exactly reproduces this experimental value at 194.7 K.

The crystal contribution was computed and integration of the isobaric heat capacity data of Giauque and Egan.[9] The highest temperature C_p reported by Giauque and Egan is at 189.78 K. The C_p at 194.7 K was linearly extrapolated to 55.51 J/mol K from the five highest temperature values reported by Giauque and Egan. A smooth integrable function for the heat capacities was created using a cubic spline in Mathematica 9.

The term $S_{gas}(T) - S_{gas}(194.7\text{ K})$ was computed according to the expressions given in Eq 13–16 using the experimental rotational constant (0.39021 cm^{-1}, which corresponds to a moment of inertia of 7.17×10^{-46} Hz)[10] and vibrational frequencies (2349, 1333, 667, & 667 cm^{-1}).[11]

Table S1: Contributions to the empirical entropy of sublimation, $\Delta S_{sub}^{emp}(T)$, in J/mol K. See Eq 19.

Temperature (K)	$S_{gas}(T)$	$S_{gas}(T) - S_{gas}(194.7\text{ K})$	$\int_{194.7K}^{T} \frac{C_{p,crystal}^{expt}(T)}{T} dT$	$\Delta S_{sub}^{emp}(T)$
15	123.67	-75.32	-68.32	122.62
20	132.04	-66.95	-67.30	129.97
30	143.84	-55.15	-63.77	138.24
40	152.21	-46.78	-59.12	141.96
50	158.70	-40.28	-54.07	143.41
60	164.01	-34.98	-49.02	143.66
70	168.49	-30.49	-44.12	143.25
80	172.38	-26.60	-39.50	142.52
90	175.81	-23.17	-35.14	141.59
100	178.89	-20.10	-31.05	140.57
110	181.67	-17.31	-27.16	139.47
120	184.23	-14.76	-23.48	138.34
130	186.59	-12.39	-19.97	137.20
150	190.88	-8.11	-13.37	134.88
170	194.71	-4.28	-7.21	132.55
190	198.20	-0.78	-1.35	130.18
194.7	198.99	0	0	129.62

4 Equation of state fitting procedures

Pressure versus volume curves were generated at several different temperatures. At a given temperature and pressure, the crystal structure was optimized by minimizing the Gibbs free energy to obtain the unit cell volume. This procedure was typically repeated for 10 (21 at 296 K) different pressures at each temperature. Sample MP2/CBS data at 296 K is plotted in Figure S1. The full set of predicted P-V data is provided in Table S3.

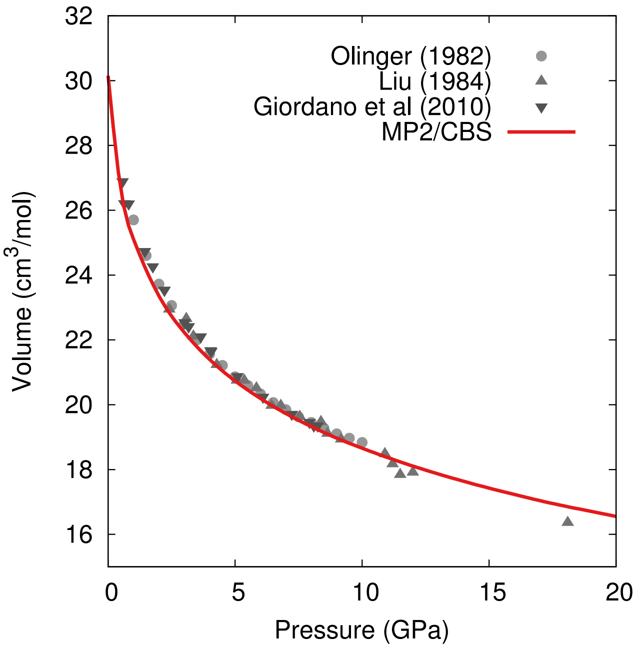

Figure S1: Comparison of the predicted and experimental pressure versus volume data at 296 K. Note that CO_2 transitions to phase III above ∼10 GPa, as seen by the sudden drop in the experimental volumes at higher pressures.

Next, the P-V data was fitted to the Vinet equation of state,[12]

$$P = 3B_0 \frac{(1-\tilde{V})}{\tilde{V}^2} \exp\left[\frac{3}{2}(B_0' - 1)(1-\tilde{V})\right] \tag{20}$$

where $\tilde{V} = (V/V_0)^{1/3}$. The constants V_0, B_0, and B_0' were obtained via non-linear least squares fitting. To explore the multitude of least squares solutions, ∼1000 fits with randomly sampled initial guess parameters spanning two-orders of magnitude were performed. For every method/basis set, the best P-V fits exhibited residuals orders of magnitude smaller than the other fits identified, and that best fit is reported here.

Note that for the Birch-Murnaghan equation of state, however, the same was not true. The Birch-Murnaghan fits proved ill-constrained, with a large number of very different parameter sets (e.g. with B_0 and B_0' values varying by up to an order of magnitude) producing fits with nearly identical residuals. Fixing the volume and linearizing the Birch-Murnaghan equation helps, but the results were extremely sensitive to the V_0 used.

Exploratory fits to the experimental data proved less well-constrained than those to the theoretical predictions due to the increased noise in the P-V data, but the parameters extracted from Monte Carlo sampling of fits to the Vinet equation exhibit much less variability than those from the Birch-Murnaghan equation. For these reasons of numerical stability, we adopted the Vinet equation.

The zero-pressure volume V_0 was treated as an adjustable fitting parameter in the Vinet equation of state. However, one could instead extract V_0 directly from the zero-pressure geometry optimization of the crystal as the given temperature, and fit only B_0 and B_0'. Table S2 compares the results from both procedures. In most cases, the resulting V_0, B_0, and B_0' values are nearly identical, with variations in the three fitted parameters of ∼0.1 or less. The largest variations between the two fitting procedures occur at 296 K, but these variations are smaller than the typical experimental uncertainties[13] in the fit parameters at the same temperature.

We also predicted the bulk modulus at 190 K using CCSD(T)/CBS. As shown in Table S2, the resulting equation of state parameters differ only marginally from the MP2/CBS ones. Given the high computational expense of performing 10 separate CCSD(T) geometry optimizations at various pressures for each temperature and the small difference between the coupled cluster and MP2 results, CCSD(T) calculations were not performed at the other temperatures.

Table S2: Comparison between Vinet EOS fits with and without constrained volumes. In the constrained volume case, the parameter V_0 was fixed to match the geometry optimized unit cell volume. In the unconstrained case, V_0 was treated as a fitting parameter.

		**Unconstrained Volume**			**Constrained Volume**		
Temperature	Method	V_0(cm^3/mol)	B_0(GPa)	B_0'	V_0(cm^3/mol)	B_0(GPa)	B_0'
No QHA	MP2/aDZ	27.0	7.8	7.8	27.0	7.8	7.8
	MP2/aTZ	25.5	9.6	7.7	25.4	9.7	7.7
	MP2/aQZ	24.9	10.2	7.7	24.9	10.4	7.7
	MP2/CBS	24.6	10.6	7.8	24.6	10.8	7.7
	Force Field	25.6	9.6	9.8			
0 K	MP2/aDZ	27.9	6.7	8.0	27.9	6.7	8.0
	MP2/aTZ	26.2	8.4	7.8	26.2	8.5	7.8
	MP2/aQZ	25.7	9.0	7.8	25.7	9.1	7.8
	MP2/CBS	25.3	9.4	7.8	25.3	9.6	7.7
	Force Field	26.5	8.1	9.8			
130 K	MP2/aDZ	29.3	4.5	8.5	29.4	4.3	8.6
	MP2/aTZ	27.3	6.1	8.2	27.4	6.0	8.3
	MP2/aQZ	26.7	6.7	8.3	26.7	6.7	8.2
	MP2/CBS	26.3	7.1	8.2	26.2	7.1	8.2
	Force Field	28.2	4.5	10.7			
190 K	MP2/aDZ	30.8	3.1	8.9	30.9	3.0	9.0
	MP2/aTZ	28.4	4.5	8.6	28.5	4.4	8.7
	MP2/aQZ	27.7	5.1	8.6	27.7	5.0	8.6
	MP2/CBS	27.2	5.5	8.5	27.2	5.5	8.5
	CCSD(T)/CBS	27.3	5.7	8.5	27.3	5.5	8.5
	Force Field	30.3	2.3	11.6			
296 K	MP2/aDZ	34.6	1.4	9.6	[a]	[a]	[a]
	MP2/aTZ	31.2	2.4	9.2	32.5	1.6	9.7
	MP2/aQZ	30.1	2.9	9.1	31.0	2.2	9.5
	MP2/CBS	29.4	3.3	9.0	30.2	2.6	9.3
	Force Field	[b]	[b]	[b]			

[a] The crystal was not bound at zero pressure and 296 K with MP2/aDZ, so V_0 could not be obtained.
[b] The crystal was not bound at zero pressure and 296 K with the force field, and the Vinet EOS fits proved numerically unstable.

Table S3: Volumes (in cm^3/mol) as a function of pressure for Vinet equation of state fitting.

Temperature	Pressure (GPa)	MP2/aDZ	MP2/aTZ	MP2/aQZ	MP2/CBS	CCSD(T)/CBS	Force Field
No QHA	0	26.98	25.44	24.91	24.59		25.57
	1	24.64	23.56	23.18	22.93		23.84
	2	23.31	22.41	22.07	21.86		22.80
	3	22.36	21.55	21.25	21.06		22.04
	4	21.62	20.88	20.61	20.44		21.45
	5	21.02	20.33	20.08	19.91		20.97
	6	20.51	19.86	19.62	19.47		20.56
	7	20.07	19.45	19.23	19.08		20.21
	8	19.69	19.10	18.88	18.75		19.90
	9	19.34	18.78	18.57	18.44		19.63
	10	19.03	18.49	18.29	18.17		19.38
0 K	0	27.89	26.23	25.67	25.31		26.46
	1	25.19	24.09	23.69	23.44		24.43
	2	23.74	22.84	22.50	22.28		23.28
	3	22.74	21.92	21.62	21.42		22.47
	4	21.96	21.21	20.94	20.77		21.83
	5	21.32	20.64	20.38	20.21		21.32
	6	20.79	20.15	19.90	19.75		20.88
	7	20.34	19.72	19.49	19.34		20.51
	8	19.94	19.35	19.13	18.98		20.19
	9	19.58	19.02	18.80	18.68		19.90
	10	19.26	18.72	18.52	18.39		19.64
130 K	0	29.42	27.36	26.68	26.25		28.12
	1	25.74	24.56	24.13	23.86		25.01
	2	24.08	23.13	22.78	22.55		23.63
	3	22.97	22.14	21.83	21.62		22.71
	4	22.13	21.38	21.09	20.91		22.01
	5	21.46	20.77	20.50	20.34		21.45
	6	20.91	20.26	20.01	19.85		20.99
	7	20.43	19.81	19.58	19.43		20.60
	8	20.02	19.42	19.20	19.06		20.26
	9	19.65	19.08	18.87	18.74		19.96
	10	19.32	18.77	18.57	18.44		19.70
190 K	0	30.94	28.53	27.72	27.19	27.31	30.30
	1	26.24	24.99	24.54	24.25	24.36	25.57
	2	24.38	23.41	23.05	22.82	22.93	23.97
	3	23.19	22.35	22.03	21.82	21.93	22.95
	4	22.31	21.55	21.26	21.07	21.18	22.20
	5	21.61	20.90	20.64	20.47	20.58	21.61
	6	21.03	20.37	20.12	19.96	20.07	21.12
	7	20.54	19.91	19.68	19.53	19.63	20.71
	8	20.12	19.51	19.29	19.15	19.25	20.36
	9	19.73	19.16	18.95	18.80	18.91	20.05
	10	19.39	18.84	18.64	18.51	18.61	19.77
296 K	0	[a]	32.50	31.05	30.15		[a]
	0.5	29.69	27.77	27.09	26.64		29.74
	1	27.36	25.94	25.44	25.11		26.99
	2	25.00	23.98	23.61	23.36		24.71
	3	23.64	22.78	22.45	22.23		23.47
	4	22.67	21.88	21.59	21.39		22.60
	5	21.91	21.18	20.91	20.74		21.93
	6	21.28	20.61	20.36	20.19		21.39
	7	20.76	20.12	19.88	19.73		20.94
	8	20.30	19.70	19.47	19.33		20.56
	9	19.91	19.33	19.11	18.97		20.23
	10	19.55	19.00	18.79	18.66		19.93
	11	19.23	18.70	18.50	18.37		19.67
	12	18.94	18.43	18.23	18.11		19.43
	13	18.68	18.17	17.99	17.86		19.21
	14	18.43	17.94	17.76	17.64		19.01
	15	18.20	17.72	17.54	17.43		[b]
	16	17.99	17.52	17.34	17.23		18.65
	17	17.79	17.33	17.16	17.04		18.49
	18	17.60	17.15	16.98	16.87		18.34
	19	17.42	16.98	16.81	16.71		18.19
	20	17.26	16.82	16.66	16.55		18.06

[a] Crystal was not bound under these conditions. [b] Optimizer had difficulty converging under these conditions.

5 Importance of phonon dispersion

As discussed in the main article and Section 1 above, lattice dynamics calculations were performed on a $3 \times 3 \times 3$ k-point grid to capture phonon dispersion. As shown in Figure S2, this proves important for capturing the proper slope of the thermal expansion. A Γ-point only phonon treatment underestimates the thermal expansion at higher temperatures.

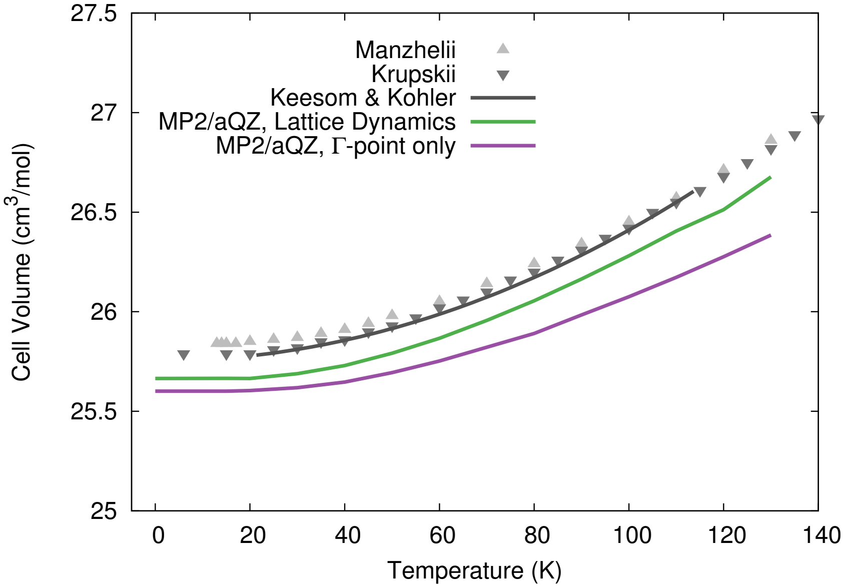

Figure S2: Comparison of the MP2/aQZ thermal expansion with Γ-point only phonons versus including phonon dispersion via lattice dynamics.

6 Intermolecular Amoeba force field parameters for CO_2

The HMBI model requires a polarizable force field for the long-range pairwise and many-body intermolecular interactions. These parameters were generated for a CO_2 using version 1.1.3 of the Poltype utility.[14] This utility first optimizes the molecular geometry at the MP2/6-31G* level of theory. Initial atomic multipole moments are calculated using Gaussian distributed multipole analysis on an MP2/6-311G** electron density. These multipole moments are subsequently refined by fitting against electrostatic potentials at various points in space computed from MP2/6-311++G(2d,2p). The atomic polarizabilities and van der Waals components are obtained via database-lookup based on atom types. See Ref 14 for details. Note that no intramolecular force field parameters (bond stretch or angle bend) are needed, since those contributions are handled quantum mechanically in the HMBI model. The final parameters used are listed below. All other parameters were set to their Amoeba defaults.

```
atom      401  401  C  "CO2"  6  12.011  2
atom      402  402  O  "CO2"  8  15.999  1

multipole 401 -402 -402  0.82686
                         0.00000  0.00000  0.00000
                         0.02680
                         0.00000  0.02680
                         0.00000  0.00000  -0.05360
multipole 402  401  402 -0.41343
                         0.00000  0.00000  -0.08355
                        -0.09705
                         0.00000 -0.09705
                         0.00000  0.00000   0.19410

polarize  401  1.3340  0.3900  402
polarize  402  0.9210  0.3900  401

vdw       401  3.8200   0.1010
vdw       402  3.4500   0.1120
```

7 Tables of predicted properties

The data tables below list the predicted properties used to generate the figures in the main paper.

7.1 Temperature-dependence of the crystal structure

The space group for phase I CO_2 is $Pa\bar{3}$. This space group is cubic so all lattice length are the same and all lattice angles are 90°. There are 4 CO_2 molecules in the unit cell. The is one carbon atom and one oxygen atom in the asymmetric unit. The carbon in the asymmetric unit is fixed at the origin. The carbon-oxygen double bond(C=O) forms a 45° angle to each of the lattice angles when projected into the sides of the unit cell. The only adjustable parameters are the length of the carbon-oxygen double bond (C=0) and the length of the unit cell (a). These values at each level of theory and basis set are presented in Table S4. The location of the remaining atoms can be determined using space group operators.

Table S4: Optimized crystal structures as a function of temperature for each level of theory. By symmetry, the entire unit cell can be constructed from the length of the C=O bond and the lattice constant *a*

	**MP2/aDZ**		**MP2/aTZ**		**MP2/aQZ**	
Temperature (K)	C=O (Å)	a (Å)	C=O (Å)	a (Å)	C=O (Å)	a (Å)
No QHA	1.17923	5.63808	1.16950	5.52818	1.16606	5.48968
0	1.17948	5.70028	1.16977	5.58498	1.16632	5.54480
15	1.17948	5.70048	1.16977	5.58501	1.16632	5.54483
20	1.17948	5.70089	1.16977	5.58501	1.16632	5.54479
30	1.17948	5.70350	1.16977	5.52818	1.16633	5.54653
40	1.17951	5.70802	1.16979	5.59040	1.16635	5.54945
50	1.17953	5.71631	1.16981	5.59529	1.16636	5.55390
60	1.17955	5.72290	1.16984	5.60121	1.16639	5.55933
70	1.17958	5.73194	1.16987	5.60846	1.16641	5.56573
80	1.17961	5.74158	1.16989	5.61626	1.16644	5.57276
90	1.17964	5.75267	1.16992	5.62481	1.16647	5.58053
100	1.17968	5.77642	1.16995	5.63355	1.16650	5.58883
110	1.17971	5.77642	1.16998	5.64299	1.16653	5.59765
120	1.17975	5.78940	1.17002	5.65314	1.16656	5.60516
130	1.17978	5.80314	1.17005	5.66428	1.16660	5.60516
150	1.17986	5.83294	1.17013	5.68859	1.16666	5.63845
170	1.17993	5.86585	1.17020	5.71587	1.16674	5.66236
190	1.18000	5.90139	1.17027	5.74419	1.16681	5.68884
194.7	1.18001	5.91014	1.17029	5.75160	1.16683	5.69548
210	1.18007	5.94031	1.17034	5.77720	1.16688	5.71844
296	[a]	[a]	1.17065	5.99905	1.16718	5.90798

	**MP2/CBS**		**CCSD(T)/CBS**		**Force Field**	
Temperature (K)	C=O (Å)	a (Å)	C=O (Å)	a (Å)	C=O (Å)	a (Å)
no QHA	1.16440	5.46627	1.16204	5.47486	1.16246	5.53796
0	1.16465	5.51930	1.16229	5.52768	1.16253	5.60147
15	1.16465	5.51932	1.16229	5.52783	1.16253	5.60162
20	1.16465	5.51951	1.16229	5.52803	1.16253	5.60208
30	1.16466	5.52083	1.16229	5.52913	1.16254	5.60455
40	1.16467	5.52361	1.16230	5.53206	1.16254	5.60922
50	1.16469	5.52779	1.16232	5.53607	1.16255	5.61584
60	1.16471	5.53305	1.16234	5.54112	1.16255	5.62403
70	1.16473	5.53866	1.16236	5.54648	1.16256	5.63358
80	1.16476	5.54517	1.16239	5.55358	1.16257	5.64436
09	1.16479	5.55239	1.16242	5.56074	1.16258	5.64436
100	1.16482	5.56005	1.16245	5.56843	1.16259	5.66930
110	1.16485	5.56908	1.16248	5.57661	1.16260	5.68329
120	1.16488	5.57696	1.16251	5.58529	1.16261	5.69892
130	1.16492	5.58638	1.16253	5.59425	1.16262	5.71594
150	1.16499	5.60602	1.16260	5.61424	1.16263	5.75417
170	1.16505	5.62798	1.16266	5.63527	1.16265	5.80052
190	1.16512	5.65246	1.16273	5.66048	1.16266	5.86032
194.7	1.16512	5.65788	1.16275	5.66668	1.16267	5.87741
210	1.16520	5.67961	1.16280	5.68744	1.16267	5.94790
296	1.16549	5.85071	[b]	[b]	[a]	[a]

[a] The crystal was not bound at zero pressure and 296 K with MP2/aDZ or the force field.
[b] The CCSD(T)/CBS structure at 296 K was not computed.

7.2 Isochoric heat capacities

Table S5: Predicted isochoric heat capacities using the quasiharmonic approximation (in units of C_V/R).

Temperature (K)	MP2/aDZ	MP2/aTZ	MP2/aQZ	MP2/CBS	Force Field
15	0.29	0.21	0.19	0.17	0.25
20	0.73	0.56	0.50	0.47	0.64
30	1.80	1.52	1.41	1.35	1.65
40	2.70	2.40	2.29	2.22	2.54
50	3.34	3.07	2.97	2.90	3.20
60	3.77	3.55	3.46	3.40	3.66
70	4.07	3.88	3.81	3.76	3.98
80	4.28	4.13	4.07	4.03	4.21
90	4.44	4.31	4.26	4.22	4.37
100	4.56	4.45	4.41	4.38	4.50
110	4.66	4.57	4.53	4.51	4.59
120	4.75	4.67	4.63	4.61	4.67
130	4.83	4.76	4.73	4.71	4.73
150	4.98	4.93	4.90	4.88	4.83
170	5.12	5.08	5.06	5.05	4.91
190	5.27	5.24	5.22	5.20	4.98

Table S6: Predicted isochoric heat capacities *without* the quasiharmonic approximation (in units of C_V/R).

Temperature (K)	MP2/aDZ	MP2/aTZ	MP2/aQZ	MP2/CBS	Force Field
15	0.22	0.16	0.14	0.13	0.16
20	0.58	0.44	0.40	0.37	0.46
30	1.55	1.30	1.21	1.16	1.32
40	2.43	2.15	2.05	1.99	2.16
50	3.08	2.83	2.73	2.67	2.81
60	3.54	3.32	3.23	3.18	3.29
70	3.86	3.68	3.60	3.56	3.64
80	4.09	3.94	3.88	3.84	3.89
90	4.27	4.14	4.09	4.06	4.08
100	4.41	4.30	4.25	4.22	4.22
110	4.52	4.42	4.38	4.36	4.33
120	4.62	4.54	4.50	4.48	4.42
130	4.71	4.64	4.60	4.59	4.49
150	4.87	4.82	4.79	4.78	4.61
170	5.03	4.99	4.97	4.95	4.70
190	5.19	5.15	5.13	5.12	4.79

7.3 Enthalpies of sublimation

Table S7: Predicted enthalpies of sublimation (in kJ/mol) using the quasiharmonic approximation

Temperature (K)	MP2/aDZ	MP2/aTZ	MP2/aQZ	MP2/CBS	CCSD(T)/CBS	Force Field
0	21.48	24.98	26.34	27.31	27.56	24.12
15	21.91	25.41	26.77	27.74	27.99	24.56
20	22.03	25.54	26.90	27.87	28.13	24.68
30	22.22	25.74	27.11	28.09	28.34	24.88
40	22.32	25.87	27.24	28.23	28.48	24.99
50	22.34	25.92	27.31	28.30	28.55	25.03
60	22.32	25.92	27.32	28.32	28.57	25.01
70	22.26	25.89	27.29	28.30	28.55	24.95
80	22.18	25.82	27.23	28.24	28.50	24.85
90	22.06	25.73	27.15	28.16	28.42	24.73
100	21.93	25.62	27.04	28.06	28.32	24.58
110	21.78	25.49	26.92	27.94	28.20	24.40
120	21.61	25.34	26.79	27.81	28.07	24.19
130	21.43	25.18	26.63	27.66	27.92	23.96
150	21.01	24.80	26.27	27.33	27.58	23.40
170	20.53	24.36	25.86	26.94	27.20	22.70
190	19.98	23.88	25.40	26.49	26.75	21.77
194.7	19.84	23.75	25.28	26.38	26.63	21.50
210	19.36	23.31	24.86	25.98	26.24	20.40

Table S8: Predicted enthalpies of sublimation (in kJ/mol) *without* the quasiharmonic approximation.

Temperature (K)	MP2/aDZ	MP2/aTZ	MP2/aQZ	MP2/CBS	CCSD(T)/CBS	Force Field
0	21.37	24.87	26.23	27.20	27.45	23.98
15	21.80	25.30	26.66	27.63	27.89	24.41
20	21.93	25.43	26.79	27.77	28.04	24.55
30	22.14	25.65	27.02	28.00	28.25	24.76
40	22.26	25.80	27.17	28.16	28.41	24.91
50	22.32	25.88	27.26	28.25	28.51	24.99
60	22.34	25.92	27.31	28.30	28.55	25.02
70	22.32	25.91	27.31	28.31	28.56	25.02
80	22.28	25.89	27.29	28.29	28.55	24.99
90	22.22	25.84	27.25	28.26	28.51	24.95
100	22.15	25.78	27.20	28.20	28.46	24.89
110	22.07	25.71	27.13	28.14	28.39	24.82
120	21.99	25.64	27.05	28.06	28.32	24.74
130	21.90	25.55	26.97	27.98	28.24	24.66
150	21.70	25.36	26.79	27.80	28.06	24.48
170	21.49	25.16	26.59	27.61	27.86	24.28
190	21.27	24.95	26.38	27.40	27.65	24.08
194.7	21.22	24.90	26.32	27.35	27.60	24.03
210	21.05	24.73	26.16	27.18	27.60	23.87

7.4 Entropies of sublimation

Table S9: Predicted entropies of sublimation (in J/mol K) using the quasiharmonic approximation. The experimentally-derived "Empirical" entropies are also listed.

Temperature (K)	MP2/aDZ	MP2/aTZ	MP2/aQZ	MP2/CBS	Force Field	Empirical
15	123.14	123.11	123.12	123.14	123.21	122.62
20	130.34	130.61	130.72	130.79	130.57	129.97
30	137.96	139.04	139.43	139.67	138.59	138.24
40	140.84	142.67	143.33	143.73	141.81	141.96
50	141.46	143.93	144.80	145.35	142.70	143.41
60	141.13	144.00	145.04	145.68	142.41	143.66
70	140.22	143.43	144.61	145.35	141.50	143.25
80	139.06	142.53	143.82	144.64	140.23	142.52
90	137.72	141.44	142.83	143.71	138.75	141.59
100	136.33	140.27	141.72	142.66	137.14	140.57
110	134.89	139.04	140.55	141.50	135.44	139.47
120	133.43	137.76	139.43	140.38	133.64	138.34
130	131.95	136.45	138.11	139.19	131.76	137.20
150	128.97	133.77	135.59	136.79	127.80	134.88
170	125.94	131.03	133.05	134.35	123.41	132.56
190	122.91	128.33	130.46	131.86	118.26	130.18
194.7	122.19	127.67	129.84	131.31	116.88	129.62
210	119.81	125.49	127.80	129.32	111.45	

Table S10: Predicted entropies of sublimation (in J/mol K) *without* the quasiharmonic approximation. The experimentally-derived "Empirical" entropies are also listed.

Temperature (K)	MP2/aDZ	MP2/aTZ	MP2/aQZ	MP2/CBS	Force Field	Empirical
15	123.27	123.19	123.20	123.20	123.39	122.62
20	130.74	130.89	130.97	131.01	131.06	129.97
30	139.08	139.92	140.22	140.39	139.97	138.24
40	142.69	144.18	144.73	145.03	144.15	141.96
50	144.06	146.05	146.78	147.20	145.97	143.41
60	144.34	146.69	147.56	148.06	146.59	143.66
70	144.08	146.68	147.66	148.22	146.58	143.25
80	143.55	146.34	147.39	147.99	146.23	142.52
90	142.88	145.81	146.92	147.55	145.71	141.59
100	142.16	145.19	146.34	147.00	145.10	140.57
110	141.41	144.52	145.71	146.38	144.45	139.47
120	140.66	143.84	145.05	145.74	143.78	138.34
130	139.93	143.15	144.39	145.09	143.11	137.20
150	138.52	141.82	143.10	143.81	141.81	134.88
170	137.21	140.56	141.86	142.58	140.59	132.56
190	136.00	139.38	140.71	141.42	139.45	130.18
194.7	135.73	139.11	140.38	141.15	139.19	129.62
210	134.87	138.27	139.63	140.33	138.38	

7.5 Bulk moduli

Tables S11 and S12 present the experimental and predicted bulk modulus values used to generate Figure 5 in the main article. Note that only DFT calculations which used a van der Waals dispersion correction (specifically, the D3(BJD) correction) were included in Figure 5.

Table S11: Bulk Modulus data for phase-I carbon dioxide.

Temperature	Method	Source	$V_0(cm^3\ mol^{-1})$	B_0(GPa)	B_0'
No QHA	MP2/aDZ	this work	27.0	7.8	7.8
	MP2/aTZ	this work	25.5	9.6	7.7
	MP2/aQZ	this work	24.9	10.2	7.7
	MP2/CBS	this work	24.6	10.6	7.8
	MP2/aDZ[a]	Li et al[15]	23.7	16.1	6.9
	MP2/aTZ	Li et al[15]	24.5	12.1	7.7
	DFT/PBE	Bonev et al[16]	31.9	3.2	8.1
	DFT/PBE	Gohr et al[17]	32.7	3.1	7.9
	DFT/PBE-D2	Gohr et al[17]	26.5	13.3	5.0
	DFT/PBE-D3(BJD)	Gohr et al[17]	27.5	9.4	5.5
	DFT/PBE-D3(ZS)	Gohr et al[17]	27.6	9.9	5.4
	DFT/PW91	Gohr et al[17]	35.4	1.3	11.5
	DFT/PW91-D3(BJD)	Gohr et al[17]	27.8	8.0	5.8
	DFT/PBE_{sol}	Gohr et al[17]	28.4	5.2	7.0
	DFT/PBE_{sol}(BJD)	Gohr et al[17]	26.5	6.8	7.2
	DFT/LDA	Gohr et al[17]	23.0	8.4	12.2
0 K	MP2/aDZ	this work	27.9	6.7	8.0
	MP2/aTZ	this work	26.2	8.4	7.8
	MP2/aQZ	this work	25.7	9.0	7.8
	MP2/CBS	this work	25.3	9.4	7.8
	DFT/LDA	Gracia et al[18]	22.2	16.6	
	Experiment	Krupskii et al[19]	25.8	7.6	
	Experiment	Zhang et al[20]		3.5	7.8
	Experiment	Trusler[21,22]	25.8	9.0	
	Experiment	Giordano et al[13]	25.8	10.4	6.8
77 K	Experiment	Liu[23b]		7.9	
130 K	MP2/aDZ	this work	29.3	4.5	8.5
	MP2/aTZ	this work	27.3	6.1	8.2
	MP2/aQZ	this work	26.7	6.7	8.3
	MP2/CBS	this work	26.3	7.1	8.2
	Experiment	Manzhelii et al[24]	26.9	5.8	
	Experiment	Krupskii et al[19]	26.8	6.9	
145 K	Experiment	Liu[23c]		4.9	
185 K	Experiment	Liu[23c]		4.2	
190 K	MP2/aDZ	this work	30.8	3.1	8.9
	MP2/aTZ	this work	28.4	4.5	8.6
	MP2/aQZ	this work	27.7	5.1	8.6
	MP2/CBS	this work	27.2	5.5	8.5
	CCSD(T)/CBS	this work	27.3	5.7	8.5
	Experiment	Krupskii et al[19]	27.9	4.0	
	Experiment	Bridgman[25]		4.8	
295–300 K	MP2/aDZ	this work	34.6	1.4	9.6
	MP2/aTZ	this work	31.2	2.4	9.2
	MP2/aQZ	this work	30.1	2.9	9.1
	MP2/CBS	this work	29.4	3.3	9.0
	DFT/LDA	Gracia et al[18]	28.3	2.8	
	Experiment	Giordano et al[13]	30.1±1	3±1	8.4±0.8
	Experiment	Giordano et al[13d]	30.7±1	2±1	9±1
	Experiment	Giordano et al[13e]	30.1±1	3±5	9±3
	Experiment	Liu[23]	31.4	2.9	7.8
	Experiment	Yoo et al[26]	25.1	6.2	6.2

[a] No counterpoise correction. [b] Re-fit of Bridgman data.[25] [c] Re-fit of Stevenson data.[27]
[d] Re-fit of Olinger data.[28] [e] Re-fit of Liu data.[23]

Table S12: Additional experimental bulk modulus data from Krupskii et al[19] and Manzhelii et al[24] for phase-I carbon dioxide.

Source	Temperature	V_0(cm^3 mol^{-1})	B_0(GPa)
Manzhelii et al	15 K	25.8	8.0
	20 K	25.84	7.94
	30 K	25.85	7.87
	40 K	25.87	7.75
	50 K	25.91	7.63
	60 K	25.98	7.41
	70 K	26.05	7.19
	80 K	26.14	6.99
	90 K	26.24	6.76
	100 K	26.45	6.54
	110 K	26.57	6.33
	120 K	26.71	6.10
	130 K	26.66	5.78
	139 K	27.01	5.46
Krupskii et al	0 K	25.80	7.593
	5 K	25.80	7.593
	10 K	25.80	7.587[a]
	15 K	25.80	7.587
	20 K	25.80	7.564
	25 K	25.80	7.536
	30 K	25.81	7.496
	35 K	25.83	7.452
	40 K	25.85	7.396
	45 K	25.88	7.342
	50 K	25.91	7.278
	55 K	25.94	7.215
	60 K	25.97	7.148
	65 K	26.04	7.082
	70 K	26.06	7.013
	75 K	26.11	6.940
	80 K	26.16	6.868
	85 K	26.21	6.798
	90 K	26.27	6.720
	95 K	26.32	6.627
	100 K	26.38	6.523
	105 K	26.44	6.414
	110 K	26.50	6.305
	115 K	26.56	6.207
	120 K	26.62	6.112
	125 K	26.69	6.161[b]
	130 K	26.76	5.907
	135 K	26.83	5.784
	140 K	26.90	5.643
	145 K	26.97	5.510
	150 K	27.05	5.362
	155 K	27.12	5.216
	160 K	27.20	5.053
	165 K	27.29	4.878
	170 K	27.37	4.690
	175 K	27.46	4.511
	180 K	27.56	4.331
	185 K	27.66	4.156
	190 K	27.76	3.971
	195 K	27.87	3.785

[a] Actual reported value of the isothermic compressibility is 1.818×10^{-11} cm^2/dyn, which corresponds to $B_0 = 5.501$ GPa and which would be a significant outlier. This appears to be a typographical error, and an isothermic compressibility of 1.318×10^{-11} cm^2/dyn ($B_0 = 7.587$ GPa) would be more reasonable.

[b] This point deviates from the monotonically decreasing B_0 and appears to be an outlier. It was omitted from Figure 5.

References

[1] G. J. O. Beran, J. Chem. Phys. **130**, 164115 (2009).

[2] G. J. O. Beran and K. Nanda, J. Phys. Chem. Lett. **1**, 3480 (2010).

[3] S. Wen and G. J. O. Beran, J. Chem. Theory Comput. **7**, 3733 (2011).

[4] K. Nanda and G. J. O. Beran, J. Chem. Phys. **137**, 174106 (2012).

[5] Y. Heit and G. J. O. Beran, J. Comp. Chem. **35**, 2205 (2014).

[6] Kaushik D. Nanda, PhD thesis, University of California, Riverside, 2013. `https://escholarship.org/uc/item/2qv949f5` Accessed August 10, 2015.

[7] S. Hirata, J. Chem. Phys. **129**, 204104 (2008).

[8] A. Otero-de-la Roza and E. R. Johnson, J. Chem. Phys. **137**, 054103 (2012).

[9] W. F. Giauque and C. J. Egan, J. Chem. Phys. **5**, 45 (1937).

[10] G. Herzberg, *Molecular Spectra and Molecular Structure III: Electronic Spectra and Electronic Structure of Polyatomic Molecules* (D. Van Nostrand Company, Inc, Princeton, New Jersey, 1966).

[11] T. Shimanouchi, *Tables of Molecular Vibrational Frequencies*, Vol. 1 of *NSRDS NBS-39* (National Bureau of Standards, Washington D.C., 1972).

[12] P. Vinet, J. R. Smith, J. Ferrante, and J. H. Rose, Phys. Rev. B **35**, 1945 (1987).

[13] V. M. Giordano, F. Datchi, F. A. Gorelli, and R. Bini, J. Chem. Phys. **133**, 144501 (2010).

[14] J. C. Wu, G. Chattree, and P. Ren, Theor. Chem. Acc. **131**, 1138 (2012).

[15] J. Li, O. Sode, and S. Hirata, J. Chem. Theory Comput. **11**, 224 (2015).

[16] S. A. Bonev, F. Gygi, T. Ogitsu, and G. Galli, Phys. Rev. Lett. **91**, 065501 (2003).

[17] S. Gohr, S. Grimme, T. Söhnel, B. Paulus, and P. Schwerdtfeger, J. Chem. Phys. **139**, 174501 (2013).

[18] L. Gracia, M. Marqués, A. Beltrán, A. M. Pendás, and J. M. Recio, J. Phys. Condens. Mat. **16**, S1263 (2004).

[19] I. N. Krupskii, A. I. Prokhvatilov, A. I. Erenburg, and A. S. Barylnik, Fiz. Nizk. Temp. **8**, 533 (1982).

[20] J. S. Zhang, S. R. Shieh, J. D. Bass, P. Dera, and V. Prakapenka, Appl. Phys. Lett. **104**, 141901 (2014).

[21] J. P. Martin Trusler, J. Phys. Chem. Ref. Data **40**, 043105 (2011).

[22] J. P. Martin Trusler, J. Phys. Chem. Ref. Data **41**, 039901 (2012).

[23] L. Liu, Earth Planet. Sci. Lett. **71**, 104 (1984).

[24] V. G. Manzhelii, A. M. Tolkachev, M. I. Bagatskii, and E. I. Voitovich, Phys. Stat. Sol. **44**, 39 (1971).

[25] P. W. Bridgman, Proc. Am. Acad. Arts Sci. **72**, 207 (1938).

[26] C. Yoo, H. Kohlmann, H. Cynn, M. Nicol, V. Iota, and T. LeBihan, Phys. Rev. B **65**, 1 (2002).

[27] R. Stevenson, J. Chem. Phys. **27**, 673 (1957).

[28] B. Olinger, J. Chem. Phys. **77**, 6255 (1982).
